# Association of screen time with attention-deficit/hyperactivity disorder symptoms and their development: the mediating role of brain structure

**DOI:** 10.1038/s41398-025-03672-1

**Published:** 2025-10-31

**Authors:** Qiulu Shou, Masatoshi Yamashita, Yoshifumi Mizuno

**Affiliations:** 1 Research Centre for Child Mental Development, University of Fukui, Fukui, Japan; 2 Division of Developmental Higher Brain Functions, United Graduate School of Child Development, University of Fukui, Fukui, Japan; 3 Life Science Innovation Centre, University of Fukui, Fukui, Japan; 4 Department of Child and Adolescent Psychological Medicine, University of Fukui Hospital, Fukui, Japan

**Keywords:** ADHD, Human behaviour, Neuroscience

## Abstract

The association among screen time, attention-deficit/hyperactivity disorder (ADHD) symptom development, and brain structure, and the neural mechanisms underlying the association between screen time and ADHD symptoms remain unclear. This study examines the relationships between the three using large-scale longitudinal data from the Adolescent Brain Cognitive Development (ABCD) study. Data on screen time, ADHD symptoms (measured via the Child Behavior Checklist), and brain structure were extracted from 10,116 children at baseline (ages 9–10) and 7880 children at a two-year follow-up. A linear mixed-effects model was used to assess the association between baseline screen time and changes in ADHD symptoms and brain structure after two years. Additionally, the mediating role of brain structure on the association between screen time and ADHD symptoms was examined. The results showed that screen time was associated with increased ADHD symptoms (β = 0.032, *p* = 0.001) and reduced cortical thickness in specific regions (right temporal pole: β = −0.036, false discovery rate (FDR)-corrected *p* = 0.020; left superior frontal gyrus: β = −0.028, FDR-corrected *p* = 0.020; and left rostral middle frontal gyrus: β = −0.030, FDR-corrected *p* = 0.020). Total cortical volume partially mediated the relationship between screen time and ADHD symptoms (β = 0.001, *p* = 0.023) at baseline. These findings suggest that screen time is associated with ADHD symptoms and brain structure, as well as their development, potentially providing insights into the neural mechanisms underlying the association between screen time and ADHD symptomatology.

## Introduction

Over the years, adolescent screen time has increased worldwide, particularly since the COVID-19 pandemic [1, 2]. Screen time influences lifestyle habits such as physical activity frequency and sleep duration [3, 4] and negatively affects mental health, behavior, and brain development [5, 6]. Given that adolescence is a critical developmental period for health, well-being, and brain maturation, shaped by both biological and environmental factors [7], some researchers recommend limiting screen time for children and adolescents while children and adolescents still have long screen time [8, 9].

Regarding the relationship between screen time and mental health, studies have found that increased screen time is associated with increased attention-deficit/hyperactivity disorder (ADHD) symptom severity [4, 5, 10–12] and a higher risk of meeting diagnostic criteria for ADHD [13], a neurodevelopmental disorder characterized by age-inappropriate inattention and/or hyperactivity/impulsivity. However, several studies have also reported that the association between screen time and ADHD tends to be weak, with small effect sizes that are not considered clinically significant or harmful [14, 15].

Previous studies have also examined the relationship between screen-based activities and brain structure [16–20]. For instance, Takeuchi et al. examined approximately 250 Japanese children and found that screen time was associated with microstructural brain development [18] and linked to smaller increases in gray and white matter volumes across brain regions, including the orbitofrontal cortex, lateral prefrontal cortex, and anterior cingulate cortex [17]. However, studies with larger samples reported no significant association between screen time and white matter microstructure abnormalities [19] or brain structures [20].

Potential mechanisms underlying the association between screen time and ADHD have been proposed, with impulsivity and sleep quality identified as mediators [4, 21]. However, little research has examined neural mediators in this relationship. One study on the role of the microstructural brain features in the link between ADHD polygenic risk and screen time [22] suggested a potential neural overlap between ADHD and screen-based activity. Additionally, ADHD has been associated with delayed cortical maturation, including alterations in cortical thickness [23] and volume [24]. Furthermore, reductions in gray matter volume across extensive brain regions have been linked to both ADHD [24] and screen-related activities [16, 17].

Despite growing interest in the impact of screen time on ADHD symptoms and brain development, most existing studies have been cross-sectional, with limited evidence on how screen time relates to changes in ADHD symptoms over time or to brain structure development. Therefore, we analyzed data from the Adolescent Brain Cognitive Development (ABCD) study, a longitudinal database comprising 11,878 children, initially aged 9–10 years, to examine the relationships. Using cross-sectional and longitudinal analyses, we investigated: [1] the association between screen time and ADHD symptoms, including their development over time; [2] the relationship between screen time and brain structure development; and [3] mediating role of brain structure in the link between screen time and ADHD symptoms and their development. To achieve this, we examined baseline and two-year follow-up data. We hypothesized that screen time is associated with ADHD symptoms and their development, and with brain structure and its development.

## Method

### Participants

The ABCD study is an ongoing, multi-center project conducted at 21 sites across the United States, following a cohort of 11,878 children initially aged 9–10 years [25]. Details regarding recruitment and ethical considerations have been previously published [26–28]. We obtained data on brain structure, behavior, and demographic background from the National Institute of Mental Health (NIMH) Data Archive ABCD Data Release 5.0. Demographic characteristics and covariates are summarized in Table 1. To maximize the sample size, all models were conducted using all available participants. For the analyses, we included data from 10,116 at baseline and 7880 at the two-year follow-up. Participants were excluded if they lacked data on brain structure, ADHD symptoms, or screen time. The specific sample size for each analysis is detailed in the “Statistical analysis” section.

**Table 1:** Demographic data and covariates of participants.

	Baseline (n = 10122)	2-year follow up (n = 7880)
Age (month)	119.00 ± 7.50	143.69 ± 7.85
Sex		
Male	5276	3740
Female	4846	4139
Parents’ income		
<49,999	2928	2090
50,000–74,999	1394	1109
75,000–99,999	1469	1207
100,000–199,999	3135	2530
≥200,000	1196	944
Parents’ education (years)	15.34 ± 2.53	15.46 ± 2.47
Race		
White	5606	4104
Black	1327	756
Hispanic	2971	1322
Asian	180	116
Other	1038	690
Pubertal status (scores)	1.63 ± 0.42	2.15 ± 0.65
Sleep duration (hours)	9.80 ± 1.26	9.32 ± 1.28
Physical activity (times per week)	3.54 ± 2.30	3.77 ± 2.16
Handedness-score rating		
Right-handed	8132	6239
Left-handed	730	558
Ambidextrous	1361	1083
Total intracranial volume (cm^3^)	1496.70 ± 143.12	1528.54 ± 146.85
Screen time (baseline) (hours)	3.70 ± 3.00	3.60 ± 2.86
ADHD T-score	53.08 ± 5.48	52.93 ± 5.13

*ADHD* attention-deficit/hyperactivity disorder.

### Screen time

Screen time was assessed using a self-reported questionnaire and calculated as the total time spent using various devices, including playing video games and watching television. The ABCD study provided screen time data separately for typical weekdays and weekends. We computed a weighted daily screen time score using the formula: 5/7× hours of screen time (weekday) + 2/7× screen time (weekend) [29]. Screen time data were available at baseline for 11,067 participants. However, the ABCD study database does not include information on screen size.

### ADHD symptoms

To assess ADHD symptoms severity, we used parent-reported ADHD-related DSM-5-oriented syndrome scales from the Child Behavior Checklist (CBCL) [30]. The dataset included 10,116 participants at baseline and 6986 at the two-year follow-up. All scores were recorded as T-scores, with higher values indicating greater behavioral problems.

Moreover, we also used the teacher-report Brief Problem Monitor (BPM) to assess teacher-reported attention problems as the sum of Q3, Q4, Q5, Q9 and Q13 as summarized in Table S1 [31, 32]. 3969 participants completed the questionnaire at baseline while 3083 participants completed the questionnaire at 2-year-follow up. However, only 813 participants completed questionnaires at both time points.

### Brain structure

Details on magnetic resonance imaging (MRI) acquisition and data preprocessing in the ABCD study have been published by Casey et al. and Hagler et al. [33, 34]. The study used 3-Tesla MRI scanners (Siemens, General Electric 750, and Philips) to obtain high-resolution T1-weighted three-dimensional structural images (1 mm isotropic), following standardized acquisition protocols [33]. Structural data were processed using FreeSurfer (version 5.3.0) with a standardized pipeline [34]. For this study, we included structural data processed using the Desikan-Killiany atlas-based classification for cortical regions and atlas-based segmentation for subcortical regions.

Only participants whose data met FreeSurfer quality control standards were included in the analysis. We examined 34 cortical regions and seven subcortical regions per hemisphere (68 and 14 regions in total) for volume and 34 cortical regions per hemisphere (68 regions in total) for cortical thickness. Additionally, as previous studies had linked ADHD to cortical gray matter volume [24, 35, 36], we included total cortical volume, as directly measured by FreeSurfer in our analyses.

### Demographic variables and covariates

Based on previous ABCD-based studies [37–40], we included the variables listed in Table 1 as covariates. Sex and race/ethnicity (White, Black, Hispanic, Asian, or other) were coded as dummy variables, while parental income was treated as a five-level categorical variable following prior research [38, 39]. We included age, parental education level, pubertal status, total intracranial volume, daily sleep duration, and physical activity as continuous variables. Parental education level was recorded by school year, consistent with previous studies [38, 39]. Pubertal status was assessed using the Pubertal Development scale [41]. Additionally, sleep duration and physical activity were included as covariates, as they are commonly considered in studies examining the relationship between screen-related activities and ADHD [3, 4, 42].

### Statistical Analysis

All statistical analyses were conducted using R, version 4.3.1 (The R Foundation for Statistical Computing, Vienna, Austria). Scatter plots were generated using R-packages “ggplot” and “ggseg” to visualize brain-related statistics.

We examined the association between screen time and ADHD symptoms using data from 10,116 participants at baseline and 6986 at the two-year follow-up. Outliers in screen time, brain structure, ADHD symptoms, and continuous covariates were winsorized at three standard deviations from the mean. For the cross-sectional analysis, we used a linear mixed-effects model with ADHD symptoms as the dependent variable, to assess the relationship between screen time and ADHD symptoms at baseline implemented via the R-package “lmertest.” Following previous studies [38, 39], family ID (denoting sibling status), multiple data collection instances, and twin or triplet status were included as random effects. The model adjusted for age, sex, race, pubertal status, household income, parental education, sleep duration, and physical activity as covariates. For the longitudinal analysis, we applied a residualized change regression model [43] to examine the relationship between changes in ADHD symptoms measured by CBCL over two years and screen time. Specifically, two-year follow-up ADHD symptoms were regressed on baseline screen time, controlling for baseline ADHD symptoms. In this model, family ID, multiple data collection instances, and twin or triplet status were again included as random effects with the same covariates. Additionally, we calculated adjusted d effect sizes for all mixed-effect regression models using the method described by Brysbaert and Stevens [44].

We further examined the association between screen time and brain structure using data from 9713 participants at baseline and 6426 at the two-year follow-up. First, we employed linear mixed-effects models to assess the relationship between screen time and brain structure development. In these models, brain structure was the dependent variable, while multiple data collection instances and twin or triplet status were included as random effects. In addition to the covariates used in the ADHD symptom analysis, we included handedness and total intracranial volume as covariates for brain volume; and handedness and mean cortical thickness as covariates for cortical thickness. To account for multiple comparisons, *p*-values were corrected using the false discovery rate (FDR) method [45]. Second, we applied the same residualized change regression model to analyze each brain structure measurement. Specifically, brain structure at the two-year follow-up was regressed on baseline screen time, controlling for baseline brain structures. As in the previous models, we included family ID, multiple data collection instances, and twin or triplet status as random effects, along with the covariates mentioned above.

Finally, we examined the mediating effect of brain structure on the association between screen time and ADHD symptoms at baseline and the development of ADHD symptoms over two years. Data from 9663 participants at baseline and 5472 at follow-up were analyzed. First, we conducted a mediation analysis to assess whether brain structures significantly associated with screen mediated the relationship between screen time and ADHD symptoms at baseline. Brain structure measures and ADHD symptoms were residualized for study site variables using a linear mixed-effects model then converted to z-scores. Mediation analysis was performed using the R-package “lanvnn” employing a standard three-variable mediation model and estimating the significance of the mediating effect via a bias-corrected bootstrap approach with 10,000 random samplings. Second, we performed a mediation analysis to assess whether changes in brain structures significantly mediated the relationship between screen time and the development of ADHD symptoms. Brain structure measures at the two-year follow-up were residualized for baseline brain structures and the above-mentioned covariates, using the linear mixed-effects model, and then converted to z-scores. ADHD symptoms at follow-up were similarly residualized for baseline ADHD symptoms and then converted to z-scores. To estimate the significance of the mediating effect, a standard three-variable mediation analysis was conducted using a bias-corrected bootstrap approach with 10,000 random samplings.

For replication analyses, we also applied the above analyses in teacher-reported attention problems calculated by BPM instead of CBCL. The results were summarized in the Supporting Information.

## Results

### Association between screen time and ADHD symptoms

**Fig. 1:**
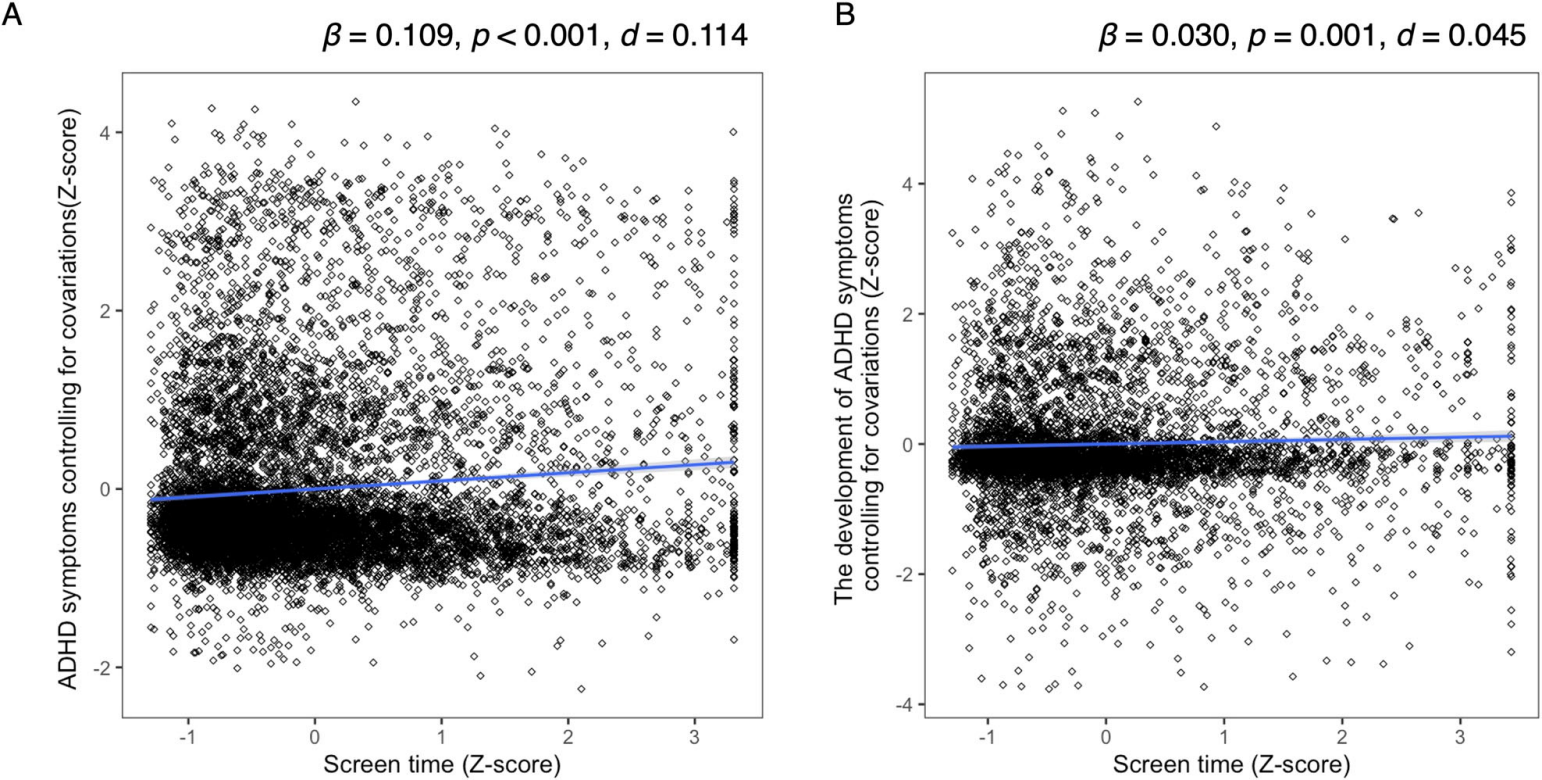
Association between screen time and ADHD symptoms and their development. **A** Relationship between screen time and ADHD symptoms. Screen time was converted to z-scores, while ADHD symptoms were adjusted for covariates using a linear mixed-effects model and converted to z-scores. **B** Relationship between screen time and ADHD symptom development. Screen time was converted to z-scores, while ADHD symptoms at the two-year follow-up were adjusted for covariates and baseline ADHD symptoms using a linear mixed-effects model and converted to z-scores. ADHD, attention-deficit/hyperactivity disorder.

We employed a linear mixed-effects regression model with ADHD symptoms as the dependent variable and screen time as the independent variable. Screen time showed a significant main effect (*β* = 0.109, 95% confidence interval [CI] = 0.088–0.130], *p* < 0.001, R^2^ = 0.049, *d* = 0.114), indicating a significant association between screen time and ADHD symptoms at baseline (Fig. 1A).

At the two-year follow-up, screen time remained a significant predicator of ADHD symptoms, even after controlling for baseline ADHD symptoms as covariates (*β* = 0.032, 95% CI = 0.021–0.089, R^2^ = 0.446, *d* = 0.045, *p* = 0.001; Fig. 1B).

### Association between screen time and brain structure

**Fig. 2:**
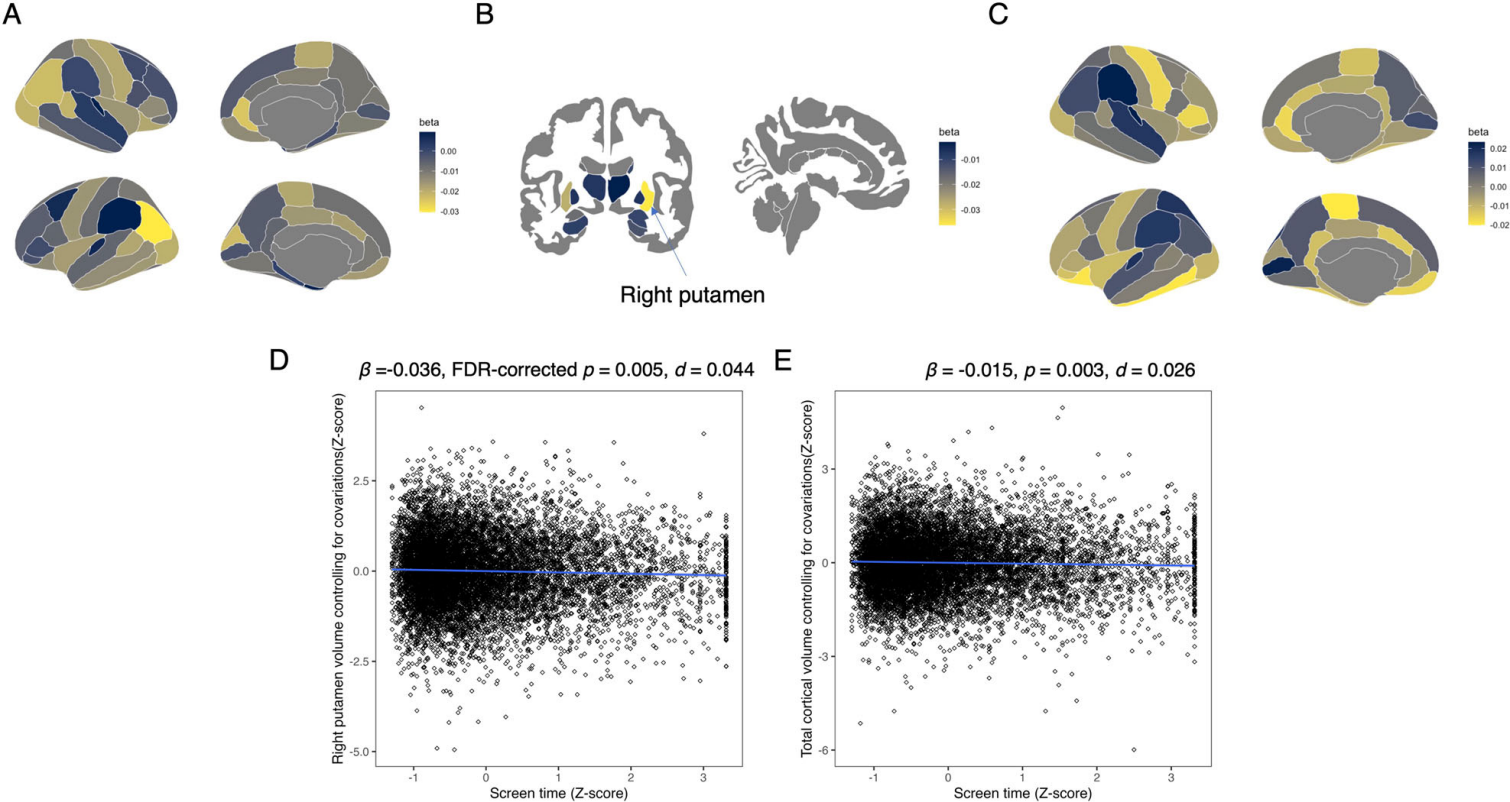
Association between screen time and brain structure at baseline. **A** Standardized coefficients (β) for the association between screen time and cortical volumes. **B** Standardized coefficients (β) for the association between screen and subcortical volumes. **C** Standardized coefficients (β) for the association between screen time and cortical thickness. **D** Association between screen time and right putamen volume. Screen time was converted to z-scores, and right putamen volume was adjusted for covariates and converted to z-scores. **E** Association between screen time and total cortical volume. Screen time was converted to z-scores, and total cortical volume was adjusted for covariates and converted to z-scores.

The association between screen time and brain volume or cortical thickness at baseline are summarized in Table S2 and Fig. 2. Screen time was negatively associated with the volume of the right putamen (*β* = −0.036, 95% CI = −0.054 to −0.019, FDR-corrected *p* = 0.005, R^2^ = 0.367, *d* = 0.044; Fig. 2D) and total cortical volume (*β* = −0.015, 95% CI = −0.025 to -0.005, *p* = 0.003, R^2^ = 0.730, *d* = 0.026; Fig. 2E).

**Fig. 3:**
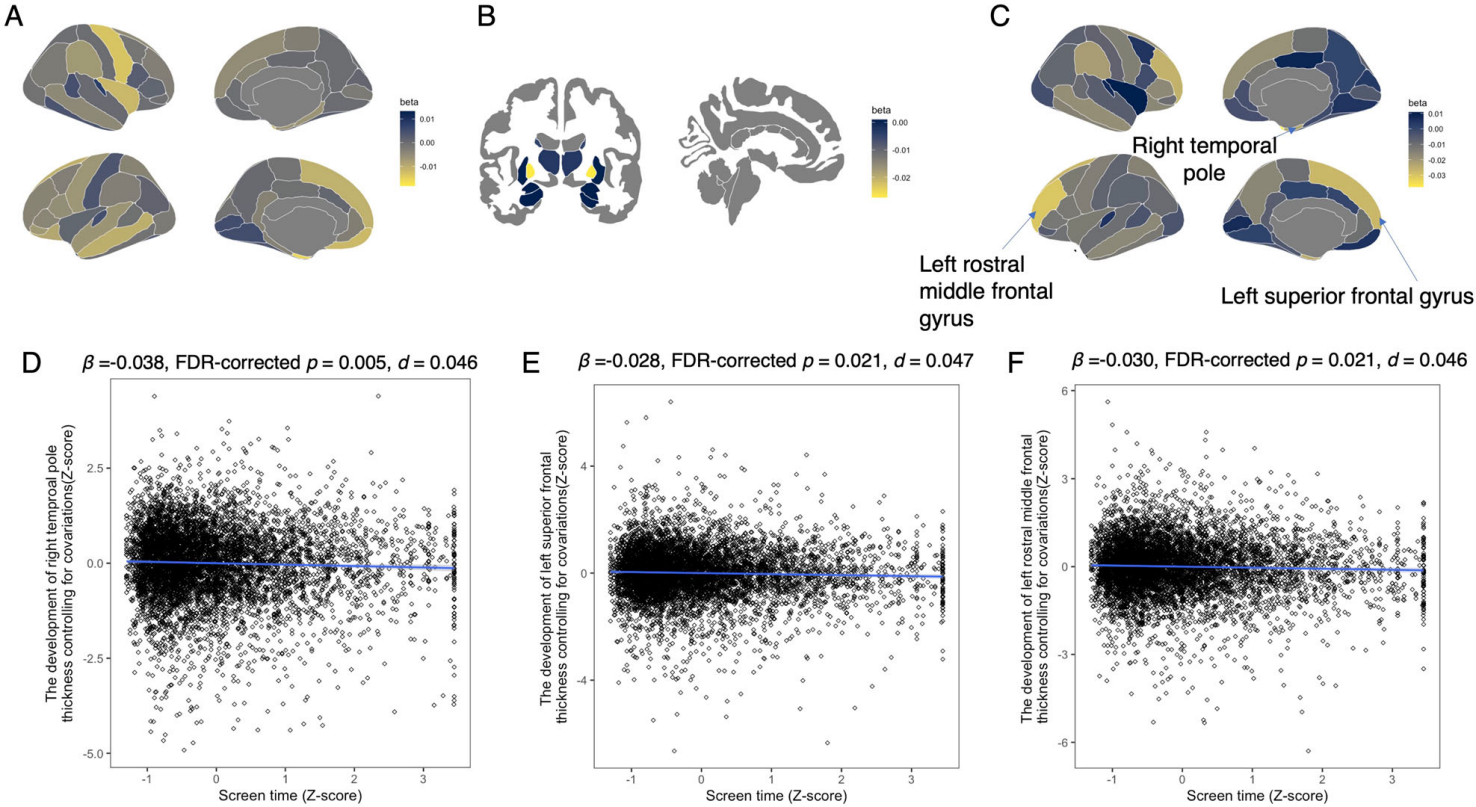
Association between screen time and brain structure development. **A** Standardized coefficients (β) for the association between screen time and cortical volume development around the cerebral cortex. **B** Standardized coefficients (β) for the association between screen time and subcortical volume development. **C** Standardized coefficients (β) for the association between screen time and cortical thickness development around the cerebral cortex. **D** Association between screen time and left temporal pole thickness. Screen time was converted to z-scores, and left temporal pole thickness was adjusted for covariates and baseline thickness, then converted to z-scores. **E** Association between screen time and right superior frontal gyrus thickness. Screen time was converted to z-scores, and right superior frontal gyrus thickness was adjusted for covariates and baseline thickness, then converted to z-scores. **F** Association between screen time and right rostral middle frontal gyrus thickness. Screen time was converted to z-scores, and right rostral middle frontal gyrus thickness was adjusted for covariates and baseline thickness, then converted to z-scores.

We then examined the association between screen time and brain structure development (Table S3 and Fig. 3). Screen time was negatively associated with cortical thicknesses in the right temporal pole (*β* = −0.038, 95% CI = −0.060 to 0.015, FDR-corrected *p* = 0.021, R^2^ = 0.333, *d* = 0.046; Fig. 3D), left superior frontal gyrus (*β* = −0.028, 95% CI = −0.044 to 0.011, FDR-corrected *p* = 0.021, R^2^ = 0.642, *d* = 0.047; Fig. 3E), and left rostral middle frontal gyrus (*β* = −0.030, 95% CI = −0.048 to −0.012, FDR-corrected *p* = 0.021, R^2^ = 0.549, *d* = 0.046; Fig. 3F).

Mediating effect of brain structure on the relationship between screen time and ADHD symptoms.

**Fig. 4:**
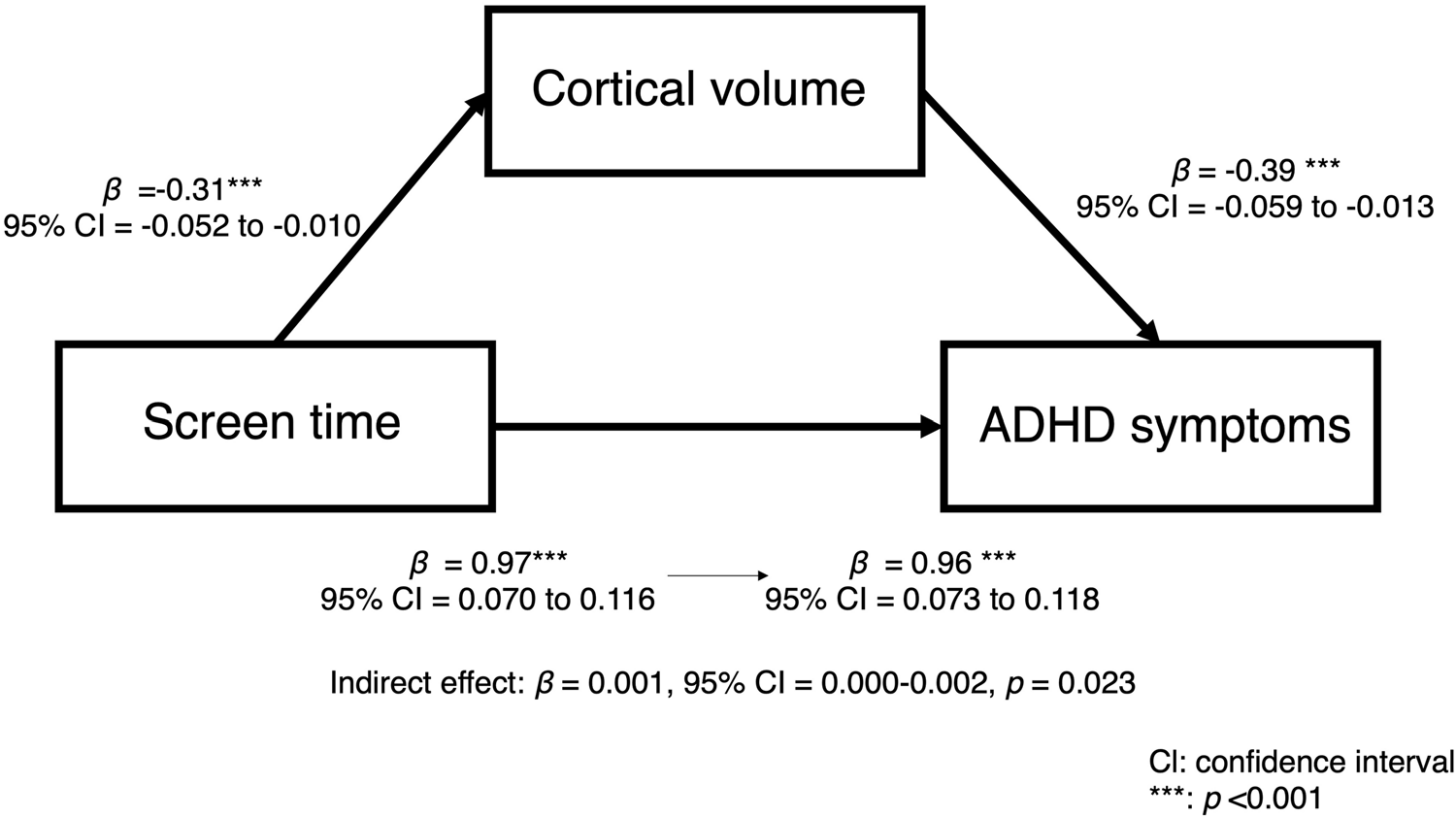
Mediating effect of total cortical volume in the association between screen time and ADHD symptoms. ADHD, attention-deficit/hyperactivity disorder.

We examined whether brain structures significantly associated with screen time at baseline mediated the relationship between screen time and ADHD symptoms. The results indicated that total cortical volume had a significant mediating effect (indirect effect *β* = 0.001, 95% CI = 0.000–0.002, *p* = 0.023; total effect: *β* = 0.088, 95% CI = 0.065 to 0.111, *p* < 0.001; Fig. 4). However, the right putamen volume did not have a mediating effect on this relationship (*β* < 0.001, 95% CI = −0.001 to 0.001, *p* = 0.889; total effect: *β* = 0.088, 95% CI = 0.066 to 0.111, *p* < 0.001).

Next, we assessed whether the development of brain structures significantly associated with screen time mediated the relationship between screen time and ADHD symptom development. No brain structure had a significant mediating effect on this relationship (right temporal pole: indirect effect: *β* < 0.001, 95% CI = −0.001 to 0.001, *p* = 0.996; total effect: *β* = 0.030, 95% CI = 0.000 to 0.006, *p* = 0.046; left superior frontal gyrus: indirect effect: *β* < 0.001, 95% CI = −0.001 to 0.002, *p* = 0.961; total effect: *β* = 0.030, 95% CI = −0.000 to 0.060, *p* = 0.045; left rostral middle frontal gyrus: indirect effect: *β* < 0.001, 95% CI = −0.001 to 0.001, *p* = 0.980; β = 0.030, 95% CI = −0.000 to 0.060, *p* = 0.047).

## Discussion

This study used a large sample from the ABCD study to examine the relationships between screen time, ADHD symptoms, and brain structure, particularly from a developmental perspective. The findings indicated that screen time was positively associated with ADHD symptoms and their development over two years. Additionally, screen time was negatively associated with right putamen volume and cortical gray matter volume at baseline, and with the development of cortical thickness in the right temporal pole, left superior frontal gyrus, and left rostral middle frontal gyrus after two years. These results align with our hypotheses, suggesting that longer screen time is associated with both ADHD symptoms and their development, as well as with brain structure and its development. Furthermore, cross-sectional analyses revealed that cortical volume partially mediated the relationship between screen time and ADHD symptoms, partially supporting the hypothesis that brain structure mediates the link between screen time and ADHD symptoms. Overall, these findings provide evidence that longer screen time is associated with increased ADHD symptoms and brain structure development, and that smaller cortical volumes may contribute to the observed negative association between screen time and ADHD symptoms.

Our results indicate that screen time is associated with ADHD symptoms and their development over time. Consistent with our findings, previous studies have reported a positive correlation between screen time and ADHD symptoms [4, 5, 10–13]. However, most studies have not specifically examined the longitudinal relationship between screen time and ADHD symptom development, leaving this association unclear [46]. By controlling for baseline ADHD symptom levels, our study provides evidence that longer screen time is associated with ADHD symptom development after two years, in children initially aged 9–10 years. This result partially aligns with the finding of Soares et al., who showed a weak association between total screen time, at ages 11, 15, and 18, and an ADHD diagnosis at the age of 22 [15]; it also extends the evidence base across a broader age range. These findings suggest that longer screen time is linked to increased ADHD symptoms in children and adolescents. In addition, the current study does not establish causality, as multiple factors could influence ADHD symptom scores on the CBCL [47]. Future research should investigate causal mechanisms. Additionally, given the small effect sizes, the clinical impact of screen time on ADHD symptoms may be marginal, and these findings should be interpreted with caution.

We examined the relationship between screen time and brain structures at baseline and the two-year follow-up. At baseline, screen time was negatively associated with right putamen volume. The putamen, a subregion of the striatum, plays a role in language processing, reward processing, cognitive function, and addiction [48]. Previous studies have linked screen time to altered functional connectivity between the frontoparietal network and the putamen [49], and internet use frequency to putamen volume changes in young women [50]; this suggests a relationship between screen-based activity and putamen structure. This association can help explain the reinforcement of screen-related behaviors, as excessive screen engagement could lead children to prefer more immediate rewards over delayed outcomes [49]. Our findings provide structural evidence that supports this explanation.

Our study comprehensively examined the relationship between screen time and brain structural development, revealing associations of screen time with the right temporal pole, left superior frontal gyrus, and left rostral middle frontal gyrus. These brain regions are involved in cognitive functions, including working memory, language processing, and attention [51–53], suggesting that screen-based activities may influence cognitive development. This finding aligns with previous studies [16, 17], that linked screen-related activity to the development of extensive cerebral cortex areas. Our study strengthens this conclusion by analyzing data from over 6000 children. Additionally, prior research found that social media use was associated with co-development patterns in key brain regions, including the bilateral superior frontal, rostral middle frontal, inferior parietal, and inferior temporal regions [54]. Our study further refines this understanding by identifying specific regions—the right temporal pole, left superior frontal gyrus, and left rostral middle frontal gyrus—that are particularly associated with screen-based activity and brain development.

Our results indicate a partial mediating effect of cortical volume in the relationship between screen time and ADHD symptoms. Specifically, longer screen time was associated with smaller cortical volume, which in turn was linked to more severe ADHD symptoms, suggesting that cortical volume may partially explain this association. Previous studies have reported reductions in cortical gray matter volume in children with ADHD [24, 36], which can be explained by a delayed cortical maturation model, where children with ADHD exhibit slower brain development compared to neurotypical peers [23]. The observed association between longer screen time and smaller cortical volume may suggest that longer screen exposure contributes to delayed brain development, potentially exacerbating ADHD symptoms. Our findings extend prior research by providing direct evidence of a mediating role of brain structure in the relationship between screen time and ADHD symptoms, suggesting a shared neural mechanism between ADHD and screen-related activity. However, as this result was derived from cross-sectional analyses of the ABCD baseline data, causality could not be established.

We also examined the mediating effect of brain structures whose development was significantly associated with screen time; however, no significant results were found. Nevertheless, as our analysis focused only on brain volume and cortical thickness, therefore, we cannot conclude that brain development is unrelated to the association between screen time and ADHD symptom development. Prior studies have linked screen time to alterations in functional brain networks [49] and microstructural brain properties [18, 22]. This suggests that these aspects of brain development may mediate the relationship between screen time and ADHD symptom development, even though our study did not identify specific brain structures that play this role. Therefore, future research should incorporate functional connectivity and microstructural brain measures to further investigate the neural mechanisms underlying screen time and ADHD symptoms.

This study is the first to examine the relationship between screen time, ADHD symptoms, and brain structure from a developmental perspective. Our findings provide evidence that longer screen time is associated with increased ADHD symptoms and brain structural development. Additionally, this study is the first to identify cortical volume as a partial mediator in the relationship between screen time and ADHD symptoms in cross-sectional analyses, suggesting that cortical volume reduction may contribute to this association. These findings enhance our understanding of the link between screen time and ADHD symptoms, as well as the neural mechanisms underlying ADHD.

## Supplementary information

Supplemental Material

## Data Availability

The Rmarkdown code used to analyze and create the manuscript is available at https://osf.io/74d5e/?view_only=ac5a3f3359bb4af3ae4086812fa8a865.
