# Supplementary material for: Association of screen time with attention-deficit/hyperactivity disorder symptoms and their development: the mediating role of brain structure
Source: Transl Psychiatry. 2025 Oct 31;15:447. doi: 10.1038/s41398-025-03672-1 (PMC12579242; doi:10.1038/s41398-025-03672-1)
Supplement: Supplementary file 1 — Supplemental Material [file 41398_2025_3672_MOESM1_ESM.docx]

**Association of Screen Time with Attention-Deficit/Hyperactivity Disorder Symptoms and Their Development: The Mediating Role of Brain Structure**

Running title: Screen Time, ADHD, and Brain Structure

Qiulu Shou (QS), PhD, Masatoshi Yamashita (MY), PhD, and Yoshifumi Mizuno (YM), MD, PhD

**Results**

**Association between screen time and teacher-reported attention problems**

We employed a linear mixed-effects regression model with teacher-reported attention problems as the dependent variable and screen time as the independent variable. Screen time showed a significant main effect (β = 0.126, 95% confidence interval [CI] = 0.091–0.162], p < 0.001, R2 = 0.049, d = 0.115; N=3,443), indicating a significant association between screen time and attention problems at baseline.

At the two-year follow-up, screen time is not associated with the change in teacher-reported attention problems (β = 0.006, 95% CI = -0.006–0.019, R2 = 0.446, d = 0.045, p =0.325; N=731).

**Mediating effect of brain structure on the relationship between screen time and attention problems**

We examined whether brain structures significantly associated with screen time at baseline mediated the relationship between screen time and attention problems. The results indicated that total cortical volume had a significant partial mediating effect (indirect effect: β = 0.003, 95% CI = 0.000 to 0.006, p = 0.049; total effect: β = 0.116, 95% CI = 0.078 to 0.154, p < 0.001) and the right putamen volume had a mediating effect on this relationship ( indirect effect: β = 0.004, 95% CI = 0.001 to 0.007, p = 0.018; total effect: β = 0.116, 95% CI = 0.078 to 0.154, p < 0.001).

**Discussion**

The result was partly consistent with the original findings. Specifically, teacher-reported attention problems have replicated the positive association between screen time and ADHD symptoms at baseline and the mediating effect of brain structures in this association. However, teacher-reported attention failed to replicate the result of the association between screen time and the development of ADHD symptoms. One possible reason we considered is the substantial decrease in sample size, since only about 800 participants completed BPM questionnaires at both time points. Moreover, previous studies reported the discrepancies between parent and teacher ratings of problem behaviors related to ADHD (De Los Reyes and Kazdin, 2005; Jungersen et al., 2021). We considered that the gap between parent-reported CBCL scores and teacher-reported BPM may lead to different results in this study.

**Reference**

De Los Reyes A, Kazdin AE. Informant discrepancies in the assessment of childhood psychopathology: A critical review, theoretical framework, and recommendations for further study. Psychol Bull. 2005;131(4):483–509.

Jungersen CM, Lonigan CJ. Do parent and teacher ratings of ADHD reflect the same constructs? A measurement invariance analysis. J Psychopathol Behav Assess. 2021;43:778–92.

| Table S1 Items used for teacher-reported ADHD symptoms | |
| --- | --- |
|  | items |
| Q3 | Fails to finish things he/she starts. |
| Q5 | Can't concentrate, can't pay attention for long. |
| Q9 | Can't sit still, restless, or hyperactive. |
| Q9 | Impulsive or acts without thinking. |
| Q13 | Inattentive or easily distracted. |

Table　S2. Results of linear mixed-effects models with screen time as the independent variable for brain structures

| Brain structure | β | 95%CI low | 95%CI　upper | t | d.f | p | FDR p | R2 | d |
| --- | --- | --- | --- | --- | --- | --- | --- | --- | --- |
| smri_vol_cdk_banksstslh | -0.019 | -0.039 | 0.002 | -1.792 | 9374.626 | 0.073 | 0.278 | 0.213 | 0.020 |
| smri_vol_cdk_cdacatelh | -0.018 | -0.039 | 0.003 | -1.649 | 9137.127 | 0.099 | 0.302 | 0.162 | 0.019 |
| smri_vol_cdk_cdmdfrlh | 0.007 | -0.013 | 0.026 | 0.677 | 9478.538 | 0.498 | 0.648 | 0.271 | 0.007 |
| smri_vol_cdk_cuneuslh | -0.023 | -0.043 | -0.003 | -2.220 | 9503.588 | 0.026 | 0.231 | 0.187 | 0.025 |
| smri_vol_cdk_ehinallh | 0.009 | -0.011 | 0.030 | 0.897 | 8192.560 | 0.370 | 0.611 | 0.182 | 0.010 |
| smri_vol_cdk_fusiformlh | -0.009 | -0.026 | 0.008 | -1.037 | 9602.141 | 0.300 | 0.572 | 0.418 | 0.011 |
| smri_vol_cdk_ifpllh | -0.030 | -0.049 | -0.012 | -3.167 | 9534.534 | 0.002 | 0.063 | 0.312 | 0.035 |
| smri_vol_cdk_iftmlh | -0.016 | -0.033 | 0.001 | -1.889 | 9616.365 | 0.059 | 0.258 | 0.410 | 0.020 |
| smri_vol_cdk_ihcatelh | -0.006 | -0.025 | 0.012 | -0.680 | 9463.861 | 0.497 | 0.648 | 0.344 | 0.008 |
| smri_vol_cdk_locclh | -0.020 | -0.038 | -0.003 | -2.279 | 9623.810 | 0.023 | 0.231 | 0.402 | 0.026 |
| smri_vol_cdk_lobfrlh | -0.014 | -0.030 | 0.002 | -1.737 | 9632.855 | 0.082 | 0.278 | 0.509 | 0.019 |
| smri_vol_cdk_linguallh | -0.010 | -0.029 | 0.009 | -1.008 | 9535.109 | 0.313 | 0.584 | 0.261 | 0.011 |
| smri_vol_cdk_mobfrlh | -0.017 | -0.033 | 0.000 | -2.000 | 9446.057 | 0.046 | 0.258 | 0.324 | 0.018 |
| smri_vol_cdk_mdtmlh | -0.014 | -0.031 | 0.003 | -1.648 | 9376.821 | 0.099 | 0.302 | 0.438 | 0.018 |
| smri_vol_cdk_parahpallh | 0.001 | -0.019 | 0.022 | 0.124 | 8861.262 | 0.902 | 0.936 | 0.175 | 0.001 |
| smri_vol_cdk_paracnlh | -0.019 | -0.038 | 0.001 | -1.883 | 9510.764 | 0.060 | 0.258 | 0.279 | 0.021 |
| smri_vol_cdk_parsopclh | -0.006 | -0.027 | 0.014 | -0.627 | 8563.691 | 0.531 | 0.669 | 0.203 | 0.007 |
| smri_vol_cdk_parsobislh | -0.002 | -0.021 | 0.017 | -0.200 | 9460.990 | 0.841 | 0.896 | 0.292 | 0.002 |
| smri_vol_cdk_parstgrislh | -0.001 | -0.021 | 0.020 | -0.056 | 8643.577 | 0.955 | 0.977 | 0.185 | 0.001 |
| smri_vol_cdk_pericclh | -0.003 | -0.024 | 0.018 | -0.271 | 9433.557 | 0.786 | 0.871 | 0.138 | 0.003 |
| smri_vol_cdk_postcnlh | -0.008 | -0.025 | 0.010 | -0.841 | 9418.321 | 0.400 | 0.631 | 0.401 | 0.009 |
| smri_vol_cdk_ptcatelh | -0.016 | -0.035 | 0.002 | -1.724 | 9183.978 | 0.085 | 0.278 | 0.346 | 0.020 |
| smri_vol_cdk_precnlh | -0.016 | -0.033 | 0.001 | -1.839 | 9305.360 | 0.066 | 0.270 | 0.438 | 0.020 |
| smri_vol_cdk_pclh | -0.005 | -0.021 | 0.012 | -0.533 | 9636.430 | 0.594 | 0.727 | 0.460 | 0.006 |
| smri_vol_cdk_rracatelh | -0.010 | -0.029 | 0.008 | -1.073 | 9627.685 | 0.283 | 0.572 | 0.347 | 0.012 |
| smri_vol_cdk_rrmdfrlh | -0.005 | -0.022 | 0.012 | -0.571 | 9656.366 | 0.568 | 0.705 | 0.419 | 0.006 |
| smri_vol_cdk_sufrlh | -0.009 | -0.025 | 0.008 | -1.039 | 9674.108 | 0.299 | 0.572 | 0.476 | 0.011 |
| smri_vol_cdk_supllh | -0.011 | -0.029 | 0.008 | -1.140 | 9632.254 | 0.254 | 0.549 | 0.347 | 0.013 |
| smri_vol_cdk_sutmlh | -0.006 | -0.023 | 0.011 | -0.731 | 9607.636 | 0.465 | 0.648 | 0.434 | 0.008 |
| smri_vol_cdk_smlh | 0.009 | -0.009 | 0.027 | 0.947 | 7614.646 | 0.344 | 0.600 | 0.359 | 0.011 |
| smri_vol_cdk_frpolelh | -0.016 | -0.036 | 0.004 | -1.569 | 9471.610 | 0.117 | 0.330 | 0.187 | 0.017 |
| smri_vol_cdk_tmpolelh | -0.007 | -0.027 | 0.014 | -0.662 | 9359.754 | 0.508 | 0.651 | 0.171 | 0.007 |
| smri_vol_cdk_trvtmlh | 0.007 | -0.013 | 0.027 | 0.707 | 9001.154 | 0.480 | 0.648 | 0.222 | 0.008 |
| smri_vol_cdk_insulalh | -0.017 | -0.033 | -0.001 | -2.023 | 9670.491 | 0.043 | 0.258 | 0.443 | 0.021 |
| smri_vol_cdk_banksstsrh | -0.021 | -0.041 | -0.002 | -2.158 | 9355.929 | 0.031 | 0.231 | 0.252 | 0.023 |
| smri_vol_cdk_cdacaterh | 0.000 | -0.021 | 0.020 | -0.044 | 8847.240 | 0.965 | 0.977 | 0.153 | 0.000 |
| smri_vol_cdk_cdmdfrrh | -0.009 | -0.028 | 0.011 | -0.892 | 9521.658 | 0.373 | 0.611 | 0.264 | 0.010 |
| smri_vol_cdk_cuneusrh | -0.010 | -0.030 | 0.009 | -1.038 | 9566.038 | 0.299 | 0.572 | 0.235 | 0.012 |
| smri_vol_cdk_ehinalrh | -0.008 | -0.029 | 0.013 | -0.764 | 9362.358 | 0.445 | 0.648 | 0.146 | 0.009 |
| smri_vol_cdk_fusiformrh | -0.015 | -0.031 | 0.002 | -1.768 | 9137.325 | 0.077 | 0.278 | 0.480 | 0.020 |
| smri_vol_cdk_ifplrh | -0.023 | -0.040 | -0.005 | -2.542 | 9592.036 | 0.011 | 0.205 | 0.385 | 0.028 |
| smri_vol_cdk_iftmrh | -0.011 | -0.028 | 0.006 | -1.287 | 9253.126 | 0.198 | 0.507 | 0.430 | 0.014 |
| smri_vol_cdk_ihcaterh | -0.008 | -0.027 | 0.011 | -0.847 | 9161.687 | 0.397 | 0.631 | 0.285 | 0.009 |
| smri_vol_cdk_loccrh | -0.021 | -0.038 | -0.004 | -2.479 | 9585.994 | 0.013 | 0.205 | 0.437 | 0.028 |
| smri_vol_cdk_lobfrrh | -0.019 | -0.034 | -0.003 | -2.372 | 9673.737 | 0.018 | 0.207 | 0.438 | 0.023 |
| smri_vol_cdk_lingualrh | -0.012 | -0.031 | 0.008 | -1.179 | 9450.308 | 0.238 | 0.543 | 0.278 | 0.013 |
| smri_vol_cdk_mobfrrh | -0.014 | -0.031 | 0.003 | -1.593 | 9637.978 | 0.111 | 0.326 | 0.406 | 0.017 |
| smri_vol_cdk_mdtmrh | -0.003 | -0.019 | 0.013 | -0.392 | 9175.667 | 0.695 | 0.814 | 0.492 | 0.004 |
| smri_vol_cdk_parahpalrh | -0.002 | -0.022 | 0.018 | -0.186 | 9533.353 | 0.853 | 0.896 | 0.204 | 0.002 |
| smri_vol_cdk_paracnrh | -0.019 | -0.039 | 0.000 | -1.924 | 9480.536 | 0.054 | 0.258 | 0.261 | 0.021 |
| smri_vol_cdk_parsopcrh | -0.002 | -0.022 | 0.018 | -0.210 | 8964.849 | 0.834 | 0.896 | 0.212 | 0.002 |
| smri_vol_cdk_parsobisrh | -0.008 | -0.028 | 0.011 | -0.821 | 9581.842 | 0.412 | 0.637 | 0.270 | 0.009 |
| smri_vol_cdk_parstgrisrh | -0.014 | -0.034 | 0.006 | -1.348 | 8869.864 | 0.178 | 0.470 | 0.190 | 0.015 |
| smri_vol_cdk_periccrh | -0.003 | -0.023 | 0.018 | -0.284 | 9454.463 | 0.776 | 0.871 | 0.156 | 0.003 |
| smri_vol_cdk_postcnrh | -0.016 | -0.034 | 0.002 | -1.754 | 9612.297 | 0.079 | 0.278 | 0.382 | 0.019 |
| smri_vol_cdk_ptcaterh | -0.012 | -0.030 | 0.007 | -1.242 | 9350.034 | 0.214 | 0.532 | 0.325 | 0.013 |
| smri_vol_cdk_precnrh | -0.019 | -0.037 | -0.002 | -2.171 | 9550.251 | 0.030 | 0.231 | 0.397 | 0.024 |
| smri_vol_cdk_pcrh | -0.009 | -0.026 | 0.007 | -1.148 | 9600.081 | 0.251 | 0.549 | 0.476 | 0.012 |
| smri_vol_cdk_rracaterh | -0.025 | -0.044 | -0.005 | -2.433 | 9196.229 | 0.015 | 0.205 | 0.238 | 0.027 |
| smri_vol_cdk_rrmdfrrh | -0.002 | -0.020 | 0.016 | -0.216 | 9669.452 | 0.829 | 0.896 | 0.376 | 0.002 |
| smri_vol_cdk_sufrrh | -0.004 | -0.021 | 0.012 | -0.499 | 9661.497 | 0.618 | 0.745 | 0.446 | 0.005 |
| smri_vol_cdk_suplrh | -0.009 | -0.027 | 0.009 | -0.956 | 9647.787 | 0.339 | 0.600 | 0.357 | 0.010 |
| smri_vol_cdk_sutmrh | 0.003 | -0.014 | 0.020 | 0.334 | 9570.159 | 0.738 | 0.841 | 0.428 | 0.003 |
| smri_vol_cdk_smrh | 0.000 | -0.019 | 0.019 | 0.009 | 8795.720 | 0.993 | 0.993 | 0.336 | 0.000 |
| smri_vol_cdk_frpolerh | -0.008 | -0.028 | 0.013 | -0.729 | 9549.176 | 0.466 | 0.648 | 0.178 | 0.008 |
| smri_vol_cdk_tmpolerh | 0.004 | -0.017 | 0.024 | 0.350 | 8704.863 | 0.726 | 0.839 | 0.164 | 0.004 |
| smri_vol_cdk_trvtmrh | 0.009 | -0.011 | 0.029 | 0.895 | 9185.746 | 0.371 | 0.611 | 0.238 | 0.010 |
| smri_vol_cdk_insularh | -0.016 | -0.032 | 0.000 | -1.901 | 9682.863 | 0.057 | 0.258 | 0.451 | 0.021 |
| smri_vol_scs_tplh | -0.007 | -0.022 | 0.008 | -0.964 | 9152.927 | 0.335 | 0.600 | 0.559 | 0.011 |
| smri_vol_scs_caudatelh | -0.017 | -0.036 | 0.001 | -1.896 | 9353.552 | 0.058 | 0.258 | 0.337 | 0.021 |
| smri_vol_scs_putamenlh | -0.028 | -0.046 | -0.010 | -2.988 | 8592.658 | 0.003 | 0.077 | 0.329 | 0.033 |
| smri_vol_scs_pallidumlh | -0.007 | -0.025 | 0.011 | -0.758 | 9348.436 | 0.448 | 0.648 | 0.328 | 0.009 |
| smri_vol_scs_hpuslh | -0.009 | -0.026 | 0.007 | -1.121 | 8881.252 | 0.262 | 0.552 | 0.478 | 0.012 |
| smri_vol_scs_amygdalalh | -0.017 | -0.034 | 0.000 | -1.969 | 9619.132 | 0.049 | 0.258 | 0.346 | 0.022 |
| smri_vol_scs_aal | -0.006 | -0.024 | 0.012 | -0.688 | 9460.302 | 0.491 | 0.648 | 0.313 | 0.007 |
| smri_vol_scs_tprh | -0.003 | -0.018 | 0.012 | -0.406 | 9520.566 | 0.685 | 0.813 | 0.570 | 0.004 |
| smri_vol_scs_caudaterh | -0.011 | -0.029 | 0.007 | -1.207 | 9250.176 | 0.227 | 0.543 | 0.352 | 0.013 |
| smri_vol_scs_putamenrh | -0.036 | -0.054 | -0.019 | -4.027 | 8446.416 | 0.000 | 0.005 | 0.367 | 0.044 |
| smri_vol_scs_pallidumrh | -0.007 | -0.025 | 0.011 | -0.773 | 9161.051 | 0.440 | 0.648 | 0.367 | 0.009 |
| smri_vol_scs_hpusrh | -0.013 | -0.029 | 0.004 | -1.474 | 8590.042 | 0.141 | 0.384 | 0.447 | 0.017 |
| smri_vol_scs_amygdalarh | -0.010 | -0.027 | 0.007 | -1.189 | 9444.161 | 0.234 | 0.543 | 0.438 | 0.013 |
| smri_vol_scs_aar | -0.007 | -0.026 | 0.012 | -0.734 | 9169.565 | 0.463 | 0.648 | 0.314 | 0.008 |
| smri_thick_cdk_banksstslh | 0.000 | -0.020 | 0.019 | -0.042 | 9506.479 | 0.966 | 0.966 | 0.268 | 0.000 |
| smri_thick_cdk_cdacatelh | -0.015 | -0.036 | 0.007 | -1.332 | 9622.131 | 0.183 | 0.522 | 0.060 | 0.015 |
| smri_thick_cdk_cdmdfrlh | 0.003 | -0.014 | 0.019 | 0.301 | 9662.371 | 0.763 | 0.868 | 0.424 | 0.003 |
| smri_thick_cdk_cuneuslh | -0.005 | -0.024 | 0.014 | -0.514 | 9690.330 | 0.607 | 0.779 | 0.305 | 0.006 |
| smri_thick_cdk_ehinallh | -0.009 | -0.031 | 0.012 | -0.855 | 9651.030 | 0.393 | 0.721 | 0.080 | 0.010 |
| smri_thick_cdk_fusiformlh | -0.015 | -0.031 | 0.002 | -1.738 | 9655.369 | 0.082 | 0.400 | 0.442 | 0.019 |
| smri_thick_cdk_ifpllh | 0.009 | -0.005 | 0.023 | 1.257 | 9638.507 | 0.209 | 0.522 | 0.553 | 0.014 |
| smri_thick_cdk_iftmlh | -0.020 | -0.037 | -0.003 | -2.285 | 9654.868 | 0.022 | 0.247 | 0.429 | 0.026 |
| smri_thick_cdk_ihcatelh | -0.012 | -0.034 | 0.009 | -1.123 | 9447.763 | 0.261 | 0.558 | 0.086 | 0.013 |
| smri_thick_cdk_locclh | -0.011 | -0.024 | 0.003 | -1.531 | 9690.348 | 0.126 | 0.475 | 0.485 | 0.015 |
| smri_thick_cdk_lobfrlh | -0.019 | -0.038 | -0.001 | -2.063 | 9658.249 | 0.039 | 0.296 | 0.321 | 0.023 |
| smri_thick_cdk_linguallh | 0.005 | -0.012 | 0.023 | 0.576 | 9683.674 | 0.565 | 0.776 | 0.333 | 0.006 |
| smri_thick_cdk_mobfrlh | -0.016 | -0.036 | 0.004 | -1.560 | 9614.874 | 0.119 | 0.475 | 0.141 | 0.017 |
| smri_thick_cdk_mdtmlh | 0.001 | -0.015 | 0.018 | 0.142 | 9586.285 | 0.887 | 0.942 | 0.410 | 0.002 |
| smri_thick_cdk_parahpallh | -0.007 | -0.028 | 0.014 | -0.639 | 9458.058 | 0.523 | 0.773 | 0.112 | 0.007 |
| smri_thick_cdk_paracnlh | -0.020 | -0.037 | -0.003 | -2.333 | 9621.000 | 0.020 | 0.247 | 0.438 | 0.027 |
| smri_thick_cdk_parsopclh | 0.006 | -0.012 | 0.023 | 0.656 | 9573.969 | 0.512 | 0.773 | 0.388 | 0.008 |
| smri_thick_cdk_parsobislh | -0.013 | -0.032 | 0.007 | -1.284 | 9522.575 | 0.199 | 0.522 | 0.264 | 0.015 |
| smri_thick_cdk_parstgrislh | -0.007 | -0.026 | 0.011 | -0.804 | 9593.912 | 0.421 | 0.735 | 0.348 | 0.009 |
| smri_thick_cdk_pericclh | 0.022 | 0.003 | 0.041 | 2.301 | 9689.693 | 0.021 | 0.247 | 0.229 | 0.025 |
| smri_thick_cdk_postcnlh | 0.003 | -0.014 | 0.020 | 0.349 | 9686.898 | 0.727 | 0.867 | 0.422 | 0.004 |
| smri_thick_cdk_ptcatelh | -0.006 | -0.027 | 0.015 | -0.554 | 9400.156 | 0.580 | 0.776 | 0.116 | 0.006 |
| smri_thick_cdk_precnlh | -0.010 | -0.025 | 0.006 | -1.220 | 9637.116 | 0.223 | 0.522 | 0.520 | 0.014 |
| smri_thick_cdk_pclh | 0.009 | -0.005 | 0.024 | 1.250 | 9655.090 | 0.211 | 0.522 | 0.543 | 0.014 |
| smri_thick_cdk_rracatelh | -0.004 | -0.024 | 0.017 | -0.364 | 9622.048 | 0.716 | 0.867 | 0.092 | 0.004 |
| smri_thick_cdk_rrmdfrlh | -0.007 | -0.022 | 0.008 | -0.911 | 9676.858 | 0.362 | 0.684 | 0.515 | 0.010 |
| smri_thick_cdk_sufrlh | 0.007 | -0.007 | 0.021 | 1.014 | 9671.331 | 0.311 | 0.621 | 0.576 | 0.011 |
| smri_thick_cdk_supllh | 0.018 | 0.004 | 0.032 | 2.601 | 9658.447 | 0.009 | 0.247 | 0.595 | 0.029 |
| smri_thick_cdk_sutmlh | 0.010 | -0.006 | 0.026 | 1.234 | 9688.331 | 0.217 | 0.522 | 0.466 | 0.014 |
| smri_thick_cdk_smlh | 0.017 | 0.002 | 0.032 | 2.183 | 9639.561 | 0.029 | 0.247 | 0.512 | 0.024 |
| smri_thick_cdk_frpolelh | 0.010 | -0.011 | 0.031 | 0.918 | 9323.103 | 0.358 | 0.684 | 0.119 | 0.010 |
| smri_thick_cdk_tmpolelh | -0.020 | -0.041 | 0.001 | -1.911 | 9623.163 | 0.056 | 0.336 | 0.173 | 0.022 |
| smri_thick_cdk_trvtmlh | 0.019 | -0.001 | 0.038 | 1.886 | 9563.819 | 0.059 | 0.336 | 0.272 | 0.022 |
| smri_thick_cdk_insulalh | -0.012 | -0.031 | 0.008 | -1.140 | 9666.732 | 0.254 | 0.558 | 0.149 | 0.012 |
| smri_thick_cdk_banksstsrh | -0.003 | -0.023 | 0.017 | -0.292 | 9562.061 | 0.771 | 0.868 | 0.244 | 0.003 |
| smri_thick_cdk_cdacaterh | 0.001 | -0.021 | 0.023 | 0.088 | 9571.182 | 0.930 | 0.966 | 0.049 | 0.001 |
| smri_thick_cdk_cdmdfrrh | -0.012 | -0.029 | 0.005 | -1.385 | 9675.692 | 0.166 | 0.522 | 0.417 | 0.016 |
| smri_thick_cdk_cuneusrh | 0.005 | -0.013 | 0.024 | 0.585 | 9678.245 | 0.559 | 0.776 | 0.329 | 0.007 |
| smri_thick_cdk_ehinalrh | 0.001 | -0.021 | 0.022 | 0.055 | 9626.207 | 0.956 | 0.966 | 0.072 | 0.001 |
| smri_thick_cdk_fusiformrh | -0.011 | -0.027 | 0.006 | -1.251 | 9605.293 | 0.211 | 0.522 | 0.439 | 0.014 |
| smri_thick_cdk_ifplrh | 0.012 | -0.002 | 0.026 | 1.665 | 9639.633 | 0.096 | 0.435 | 0.572 | 0.019 |
| smri_thick_cdk_iftmrh | 0.000 | -0.016 | 0.017 | 0.046 | 9654.623 | 0.963 | 0.966 | 0.440 | 0.001 |
| smri_thick_cdk_ihcaterh | -0.009 | -0.031 | 0.013 | -0.820 | 9309.838 | 0.412 | 0.735 | 0.064 | 0.009 |
| smri_thick_cdk_loccrh | -0.009 | -0.023 | 0.004 | -1.400 | 9692.265 | 0.162 | 0.522 | 0.521 | 0.014 |
| smri_thick_cdk_lobfrrh | -0.006 | -0.025 | 0.012 | -0.685 | 9646.303 | 0.493 | 0.762 | 0.263 | 0.007 |
| smri_thick_cdk_lingualrh | -0.005 | -0.023 | 0.013 | -0.534 | 9686.160 | 0.593 | 0.776 | 0.350 | 0.006 |
| smri_thick_cdk_mobfrrh | -0.007 | -0.027 | 0.013 | -0.712 | 9645.455 | 0.477 | 0.762 | 0.173 | 0.008 |
| smri_thick_cdk_mdtmrh | 0.004 | -0.011 | 0.020 | 0.539 | 9666.941 | 0.590 | 0.776 | 0.438 | 0.006 |
| smri_thick_cdk_parahpalrh | -0.005 | -0.025 | 0.016 | -0.472 | 9536.708 | 0.637 | 0.802 | 0.154 | 0.005 |
| smri_thick_cdk_paracnrh | -0.013 | -0.030 | 0.005 | -1.439 | 9634.771 | 0.150 | 0.522 | 0.427 | 0.016 |
| smri_thick_cdk_parsopcrh | 0.003 | -0.015 | 0.021 | 0.322 | 9568.476 | 0.747 | 0.868 | 0.335 | 0.004 |
| smri_thick_cdk_parsobisrh | -0.002 | -0.021 | 0.017 | -0.216 | 9458.497 | 0.829 | 0.895 | 0.260 | 0.002 |
| smri_thick_cdk_parstgrisrh | -0.017 | -0.035 | 0.001 | -1.834 | 9575.603 | 0.067 | 0.349 | 0.359 | 0.021 |
| smri_thick_cdk_periccrh | 0.012 | -0.007 | 0.032 | 1.266 | 9694.133 | 0.206 | 0.522 | 0.216 | 0.014 |
| smri_thick_cdk_postcnrh | -0.002 | -0.020 | 0.015 | -0.258 | 9670.442 | 0.796 | 0.874 | 0.346 | 0.003 |
| smri_thick_cdk_ptcaterh | -0.008 | -0.029 | 0.014 | -0.701 | 9535.111 | 0.483 | 0.762 | 0.124 | 0.008 |
| smri_thick_cdk_precnrh | -0.017 | -0.034 | 0.000 | -1.937 | 9372.105 | 0.053 | 0.336 | 0.412 | 0.022 |
| smri_thick_cdk_pcrh | 0.008 | -0.007 | 0.023 | 1.016 | 9654.016 | 0.310 | 0.621 | 0.553 | 0.011 |
| smri_thick_cdk_rracaterh | -0.017 | -0.038 | 0.004 | -1.569 | 9629.887 | 0.117 | 0.475 | 0.075 | 0.018 |
| smri_thick_cdk_rrmdfrrh | -0.005 | -0.020 | 0.011 | -0.570 | 9674.607 | 0.569 | 0.776 | 0.468 | 0.006 |
| smri_thick_cdk_sufrrh | 0.002 | -0.012 | 0.016 | 0.281 | 9682.875 | 0.779 | 0.868 | 0.523 | 0.003 |
| smri_thick_cdk_suplrh | 0.006 | -0.009 | 0.020 | 0.784 | 9667.081 | 0.433 | 0.736 | 0.586 | 0.009 |
| smri_thick_cdk_sutmrh | 0.018 | 0.002 | 0.034 | 2.186 | 9684.403 | 0.029 | 0.247 | 0.495 | 0.025 |
| smri_thick_cdk_smrh | 0.023 | 0.008 | 0.038 | 2.999 | 9603.384 | 0.003 | 0.184 | 0.454 | 0.032 |
| smri_thick_cdk_frpolerh | -0.008 | -0.029 | 0.013 | -0.705 | 9171.307 | 0.481 | 0.762 | 0.115 | 0.008 |
| smri_thick_cdk_tmpolerh | -0.012 | -0.032 | 0.009 | -1.121 | 9618.735 | 0.262 | 0.558 | 0.156 | 0.013 |
| smri_thick_cdk_trvtmrh | 0.023 | 0.004 | 0.043 | 2.381 | 9313.188 | 0.017 | 0.247 | 0.229 | 0.026 |
| smri_thick_cdk_insularh | -0.004 | -0.024 | 0.016 | -0.394 | 9646.210 | 0.693 | 0.857 | 0.148 | 0.004 |

CI, confidence interval

The codes of the brain structures can be checked at the ABCD study website: https://nda.nih.gov/data_structure.html?short_name=abcd_smrip101

Table S3. Results of linear mixed-effects models with screen time as the independent variable for the development of brain structures

| Brain structure | β | 95%CI low | 95%CI　upper | t | d.f | p | FDR p | R2 | d |
| --- | --- | --- | --- | --- | --- | --- | --- | --- | --- |
| smri_vol_cdk_banksstslh | -0.001 | -0.009 | 0.008 | -0.121 | 6030.450 | 0.904 | 0.978 | 0.904 | 0.002 |
| smri_vol_cdk_cdacatelh | 0.001 | -0.006 | 0.007 | 0.237 | 6234.528 | 0.812 | 0.978 | 0.948 | 0.003 |
| smri_vol_cdk_cdmdfrlh | -0.003 | -0.011 | 0.005 | -0.706 | 5990.453 | 0.480 | 0.914 | 0.919 | 0.010 |
| smri_vol_cdk_cuneuslh | -0.001 | -0.008 | 0.006 | -0.264 | 6190.696 | 0.792 | 0.978 | 0.939 | 0.004 |
| smri_vol_cdk_ehinallh | -0.016 | -0.030 | -0.001 | -2.154 | 6135.681 | 0.031 | 0.427 | 0.729 | 0.030 |
| smri_vol_cdk_fusiformlh | 0.003 | -0.004 | 0.011 | 0.953 | 6134.781 | 0.341 | 0.798 | 0.934 | 0.013 |
| smri_vol_cdk_ifpllh | 0.002 | -0.004 | 0.008 | 0.518 | 5893.833 | 0.605 | 0.929 | 0.951 | 0.007 |
| smri_vol_cdk_iftmlh | -0.001 | -0.007 | 0.006 | -0.284 | 6253.278 | 0.776 | 0.978 | 0.945 | 0.004 |
| smri_vol_cdk_ihcatelh | -0.004 | -0.011 | 0.003 | -0.994 | 6242.563 | 0.320 | 0.795 | 0.935 | 0.014 |
| smri_vol_cdk_locclh | -0.003 | -0.009 | 0.004 | -0.802 | 6251.284 | 0.422 | 0.863 | 0.946 | 0.011 |
| smri_vol_cdk_lobfrlh | -0.009 | -0.018 | 0.000 | -1.927 | 6133.223 | 0.054 | 0.523 | 0.886 | 0.027 |
| smri_vol_cdk_linguallh | 0.005 | -0.001 | 0.011 | 1.773 | 6183.930 | 0.076 | 0.523 | 0.955 | 0.025 |
| smri_vol_cdk_mobfrlh | -0.012 | -0.025 | 0.001 | -1.797 | 6091.656 | 0.072 | 0.523 | 0.775 | 0.025 |
| smri_vol_cdk_mdtmlh | -0.010 | -0.017 | -0.004 | -3.013 | 6097.293 | 0.003 | 0.102 | 0.940 | 0.042 |
| smri_vol_cdk_parahpallh | -0.004 | -0.014 | 0.007 | -0.663 | 6186.864 | 0.507 | 0.914 | 0.850 | 0.009 |
| smri_vol_cdk_paracnlh | -0.001 | -0.010 | 0.008 | -0.219 | 5968.410 | 0.827 | 0.978 | 0.884 | 0.003 |
| smri_vol_cdk_parsopclh | -0.005 | -0.013 | 0.002 | -1.396 | 6225.815 | 0.163 | 0.636 | 0.922 | 0.020 |
| smri_vol_cdk_parsobislh | -0.007 | -0.016 | 0.002 | -1.573 | 6042.323 | 0.116 | 0.563 | 0.895 | 0.022 |
| smri_vol_cdk_parstgrislh | -0.004 | -0.011 | 0.003 | -1.129 | 6050.615 | 0.259 | 0.708 | 0.931 | 0.016 |
| smri_vol_cdk_pericclh | 0.005 | -0.003 | 0.013 | 1.296 | 6218.674 | 0.195 | 0.663 | 0.925 | 0.018 |
| smri_vol_cdk_postcnlh | 0.004 | -0.004 | 0.011 | 0.974 | 6203.647 | 0.330 | 0.796 | 0.931 | 0.014 |
| smri_vol_cdk_ptcatelh | -0.001 | -0.007 | 0.006 | -0.183 | 6119.738 | 0.855 | 0.978 | 0.948 | 0.003 |
| smri_vol_cdk_precnlh | -0.007 | -0.014 | 0.001 | -1.764 | 6019.491 | 0.078 | 0.523 | 0.925 | 0.025 |
| smri_vol_cdk_pclh | 0.000 | -0.007 | 0.007 | 0.059 | 6024.041 | 0.953 | 0.978 | 0.932 | 0.001 |
| smri_vol_cdk_rracatelh | -0.003 | -0.012 | 0.006 | -0.639 | 6248.330 | 0.523 | 0.914 | 0.884 | 0.009 |
| smri_vol_cdk_rrmdfrlh | -0.004 | -0.012 | 0.005 | -0.891 | 6025.088 | 0.373 | 0.835 | 0.902 | 0.012 |
| smri_vol_cdk_sufrlh | -0.011 | -0.018 | -0.003 | -2.902 | 5951.264 | 0.004 | 0.102 | 0.932 | 0.041 |
| smri_vol_cdk_supllh | -0.004 | -0.013 | 0.005 | -0.846 | 6119.086 | 0.397 | 0.835 | 0.890 | 0.012 |
| smri_vol_cdk_sutmlh | -0.007 | -0.013 | 0.000 | -2.050 | 6136.334 | 0.040 | 0.473 | 0.948 | 0.029 |
| smri_vol_cdk_smlh | -0.003 | -0.010 | 0.004 | -0.787 | 6144.948 | 0.431 | 0.863 | 0.930 | 0.011 |
| smri_vol_cdk_frpolelh | -0.018 | -0.034 | -0.003 | -2.302 | 6053.483 | 0.021 | 0.351 | 0.679 | 0.032 |
| smri_vol_cdk_tmpolelh | 0.013 | -0.004 | 0.030 | 1.551 | 5995.184 | 0.121 | 0.563 | 0.623 | 0.022 |
| smri_vol_cdk_trvtmlh | 0.005 | -0.003 | 0.013 | 1.172 | 6114.117 | 0.241 | 0.682 | 0.911 | 0.017 |
| smri_vol_cdk_insulalh | 0.001 | -0.011 | 0.013 | 0.156 | 6244.520 | 0.876 | 0.978 | 0.802 | 0.002 |
| smri_vol_cdk_banksstsrh | 0.005 | -0.004 | 0.013 | 1.081 | 5765.753 | 0.280 | 0.728 | 0.909 | 0.015 |
| smri_vol_cdk_cdacaterh | -0.001 | -0.008 | 0.005 | -0.395 | 6023.556 | 0.693 | 0.978 | 0.939 | 0.006 |
| smri_vol_cdk_cdmdfrrh | -0.004 | -0.014 | 0.005 | -0.849 | 5795.584 | 0.396 | 0.835 | 0.876 | 0.012 |
| smri_vol_cdk_cuneusrh | 0.001 | -0.007 | 0.009 | 0.266 | 6140.558 | 0.790 | 0.978 | 0.920 | 0.004 |
| smri_vol_cdk_ehinalrh | -0.006 | -0.021 | 0.008 | -0.861 | 5762.261 | 0.389 | 0.835 | 0.717 | 0.012 |
| smri_vol_cdk_fusiformrh | 0.002 | -0.005 | 0.009 | 0.542 | 6092.013 | 0.588 | 0.929 | 0.938 | 0.008 |
| smri_vol_cdk_ifplrh | -0.002 | -0.009 | 0.004 | -0.686 | 5492.018 | 0.493 | 0.914 | 0.944 | 0.010 |
| smri_vol_cdk_iftmrh | 0.004 | -0.003 | 0.010 | 1.174 | 6053.533 | 0.241 | 0.682 | 0.947 | 0.016 |
| smri_vol_cdk_ihcaterh | -0.001 | -0.008 | 0.007 | -0.145 | 6201.682 | 0.884 | 0.978 | 0.917 | 0.002 |
| smri_vol_cdk_loccrh | -0.001 | -0.007 | 0.005 | -0.289 | 6104.106 | 0.773 | 0.978 | 0.949 | 0.004 |
| smri_vol_cdk_lobfrrh | 0.000 | -0.012 | 0.011 | -0.038 | 6232.684 | 0.970 | 0.978 | 0.815 | 0.001 |
| smri_vol_cdk_lingualrh | 0.000 | -0.006 | 0.006 | 0.066 | 5775.533 | 0.947 | 0.978 | 0.954 | 0.001 |
| smri_vol_cdk_mobfrrh | 0.000 | -0.013 | 0.012 | -0.028 | 6213.966 | 0.978 | 0.978 | 0.797 | 0.000 |
| smri_vol_cdk_mdtmrh | -0.005 | -0.011 | 0.001 | -1.526 | 6228.496 | 0.127 | 0.563 | 0.949 | 0.022 |
| smri_vol_cdk_parahpalrh | -0.006 | -0.018 | 0.005 | -1.071 | 6389.159 | 0.284 | 0.728 | 0.814 | 0.015 |
| smri_vol_cdk_paracnrh | -0.003 | -0.013 | 0.007 | -0.620 | 6096.657 | 0.535 | 0.914 | 0.877 | 0.009 |
| smri_vol_cdk_parsopcrh | 0.003 | -0.007 | 0.012 | 0.507 | 5819.148 | 0.612 | 0.929 | 0.874 | 0.007 |
| smri_vol_cdk_parsobisrh | -0.001 | -0.009 | 0.008 | -0.146 | 6034.520 | 0.884 | 0.978 | 0.904 | 0.002 |
| smri_vol_cdk_parstgrisrh | 0.001 | -0.008 | 0.010 | 0.250 | 5922.353 | 0.803 | 0.978 | 0.892 | 0.003 |
| smri_vol_cdk_periccrh | -0.002 | -0.010 | 0.006 | -0.561 | 6135.818 | 0.575 | 0.929 | 0.915 | 0.008 |
| smri_vol_cdk_postcnrh | 0.000 | -0.008 | 0.008 | -0.028 | 6158.411 | 0.978 | 0.978 | 0.918 | 0.000 |
| smri_vol_cdk_ptcaterh | 0.000 | -0.006 | 0.006 | 0.073 | 6069.529 | 0.942 | 0.978 | 0.952 | 0.001 |
| smri_vol_cdk_precnrh | -0.013 | -0.023 | -0.003 | -2.654 | 6002.113 | 0.008 | 0.164 | 0.873 | 0.037 |
| smri_vol_cdk_pcrh | 0.000 | -0.008 | 0.008 | -0.031 | 6108.820 | 0.975 | 0.978 | 0.921 | 0.000 |
| smri_vol_cdk_rracaterh | 0.000 | -0.009 | 0.009 | -0.044 | 6197.890 | 0.965 | 0.978 | 0.897 | 0.001 |
| smri_vol_cdk_rrmdfrrh | -0.003 | -0.012 | 0.006 | -0.625 | 6039.277 | 0.532 | 0.914 | 0.883 | 0.009 |
| smri_vol_cdk_sufrrh | -0.005 | -0.014 | 0.003 | -1.191 | 5940.012 | 0.234 | 0.682 | 0.903 | 0.017 |
| smri_vol_cdk_suplrh | 0.000 | -0.011 | 0.010 | -0.029 | 6155.920 | 0.977 | 0.978 | 0.855 | 0.000 |
| smri_vol_cdk_sutmrh | 0.000 | -0.006 | 0.007 | 0.153 | 6210.150 | 0.879 | 0.978 | 0.947 | 0.002 |
| smri_vol_cdk_smrh | -0.007 | -0.017 | 0.002 | -1.486 | 6179.083 | 0.137 | 0.563 | 0.881 | 0.021 |
| smri_vol_cdk_frpolerh | 0.005 | -0.012 | 0.021 | 0.563 | 6225.599 | 0.573 | 0.929 | 0.640 | 0.008 |
| smri_vol_cdk_tmpolerh | -0.011 | -0.028 | 0.005 | -1.347 | 5806.042 | 0.178 | 0.663 | 0.640 | 0.019 |
| smri_vol_cdk_trvtmrh | 0.008 | -0.001 | 0.017 | 1.664 | 5957.520 | 0.096 | 0.563 | 0.888 | 0.023 |
| smri_vol_cdk_insularh | -0.012 | -0.025 | 0.002 | -1.734 | 6229.075 | 0.083 | 0.523 | 0.772 | 0.024 |
| smri_vol_scs_tplh | -0.010 | -0.022 | 0.002 | -1.598 | 5979.156 | 0.110 | 0.563 | 0.802 | 0.022 |
| smri_vol_scs_caudatelh | -0.004 | -0.011 | 0.002 | -1.283 | 6006.808 | 0.200 | 0.663 | 0.941 | 0.018 |
| smri_vol_scs_putamenlh | -0.006 | -0.016 | 0.003 | -1.275 | 6384.839 | 0.202 | 0.663 | 0.868 | 0.018 |
| smri_vol_scs_pallidumlh | -0.016 | -0.034 | 0.002 | -1.758 | 6143.669 | 0.079 | 0.523 | 0.520 | 0.024 |
| smri_vol_scs_hpuslh | -0.003 | -0.012 | 0.006 | -0.672 | 5957.241 | 0.502 | 0.914 | 0.889 | 0.009 |
| smri_vol_scs_amygdalalh | -0.003 | -0.017 | 0.010 | -0.470 | 6026.653 | 0.638 | 0.951 | 0.736 | 0.007 |
| smri_vol_scs_aal | 0.003 | -0.014 | 0.020 | 0.368 | 6047.388 | 0.713 | 0.978 | 0.601 | 0.005 |
| smri_vol_scs_tprh | -0.003 | -0.014 | 0.008 | -0.535 | 6109.925 | 0.593 | 0.929 | 0.840 | 0.007 |
| smri_vol_scs_caudaterh | -0.005 | -0.011 | 0.002 | -1.487 | 5970.898 | 0.137 | 0.563 | 0.943 | 0.021 |
| smri_vol_scs_putamenrh | 0.001 | -0.009 | 0.010 | 0.120 | 5683.909 | 0.905 | 0.978 | 0.890 | 0.002 |
| smri_vol_scs_pallidumrh | -0.027 | -0.044 | -0.011 | -3.217 | 5922.772 | 0.001 | 0.102 | 0.630 | 0.045 |
| smri_vol_scs_hpusrh | 0.001 | -0.007 | 0.009 | 0.164 | 6124.882 | 0.870 | 0.978 | 0.915 | 0.002 |
| smri_vol_scs_amygdalarh | 0.001 | -0.012 | 0.014 | 0.158 | 6170.174 | 0.875 | 0.978 | 0.787 | 0.002 |
| smri_vol_scs_aar | -0.009 | -0.024 | 0.006 | -1.196 | 5734.026 | 0.232 | 0.682 | 0.708 | 0.017 |
| smri_thick_cdk_banksstslh | -0.003 | -0.016 | 0.009 | -0.524 | 5916.709 | 0.600 | 0.785 | 0.775 | 0.007 |
| smri_thick_cdk_cdacatelh | 0.000 | -0.013 | 0.014 | 0.053 | 6255.788 | 0.958 | 0.972 | 0.747 | 0.001 |
| smri_thick_cdk_cdmdfrlh | -0.013 | -0.031 | 0.004 | -1.498 | 6208.957 | 0.134 | 0.415 | 0.585 | 0.021 |
| smri_thick_cdk_cuneuslh | -0.003 | -0.016 | 0.010 | -0.448 | 6257.596 | 0.654 | 0.802 | 0.764 | 0.006 |
| smri_thick_cdk_ehinallh | -0.025 | -0.045 | -0.005 | -2.452 | 6108.011 | 0.014 | 0.171 | 0.450 | 0.033 |
| smri_thick_cdk_fusiformlh | -0.004 | -0.020 | 0.013 | -0.439 | 6204.135 | 0.660 | 0.802 | 0.654 | 0.006 |
| smri_thick_cdk_ifpllh | -0.009 | -0.026 | 0.008 | -1.026 | 6138.406 | 0.305 | 0.575 | 0.613 | 0.014 |
| smri_thick_cdk_iftmlh | -0.012 | -0.029 | 0.004 | -1.500 | 6201.302 | 0.134 | 0.415 | 0.648 | 0.021 |
| smri_thick_cdk_ihcatelh | -0.006 | -0.019 | 0.006 | -0.957 | 6145.399 | 0.338 | 0.575 | 0.792 | 0.013 |
| smri_thick_cdk_locclh | -0.003 | -0.017 | 0.010 | -0.493 | 6316.488 | 0.622 | 0.798 | 0.733 | 0.007 |
| smri_thick_cdk_lobfrlh | -0.011 | -0.030 | 0.008 | -1.105 | 6238.971 | 0.269 | 0.572 | 0.515 | 0.015 |
| smri_thick_cdk_linguallh | 0.001 | -0.012 | 0.015 | 0.173 | 6238.950 | 0.863 | 0.946 | 0.756 | 0.002 |
| smri_thick_cdk_mobfrlh | 0.006 | -0.015 | 0.026 | 0.536 | 6231.604 | 0.592 | 0.785 | 0.447 | 0.008 |
| smri_thick_cdk_mdtmlh | -0.016 | -0.032 | -0.001 | -2.039 | 6244.174 | 0.041 | 0.256 | 0.646 | 0.028 |
| smri_thick_cdk_parahpallh | -0.004 | -0.015 | 0.007 | -0.744 | 5558.544 | 0.457 | 0.678 | 0.835 | 0.010 |
| smri_thick_cdk_paracnlh | -0.009 | -0.025 | 0.006 | -1.208 | 6252.206 | 0.227 | 0.498 | 0.683 | 0.017 |
| smri_thick_cdk_parsopclh | -0.013 | -0.029 | 0.003 | -1.551 | 6233.664 | 0.121 | 0.411 | 0.638 | 0.021 |
| smri_thick_cdk_parsobislh | -0.005 | -0.021 | 0.011 | -0.650 | 6284.185 | 0.516 | 0.702 | 0.667 | 0.009 |
| smri_thick_cdk_parstgrislh | -0.012 | -0.029 | 0.005 | -1.428 | 6171.655 | 0.153 | 0.434 | 0.616 | 0.020 |
| smri_thick_cdk_pericclh | 0.007 | -0.007 | 0.022 | 0.973 | 6242.499 | 0.331 | 0.575 | 0.698 | 0.013 |
| smri_thick_cdk_postcnlh | -0.007 | -0.022 | 0.007 | -0.985 | 6307.602 | 0.325 | 0.575 | 0.711 | 0.014 |
| smri_thick_cdk_ptcatelh | 0.001 | -0.012 | 0.014 | 0.105 | 6376.432 | 0.916 | 0.958 | 0.776 | 0.001 |
| smri_thick_cdk_precnlh | -0.014 | -0.031 | 0.004 | -1.555 | 6240.151 | 0.120 | 0.411 | 0.575 | 0.022 |
| smri_thick_cdk_pclh | -0.016 | -0.031 | -0.001 | -2.129 | 6175.405 | 0.033 | 0.227 | 0.717 | 0.030 |
| smri_thick_cdk_rracatelh | -0.010 | -0.030 | 0.009 | -1.020 | 6294.475 | 0.308 | 0.575 | 0.452 | 0.014 |
| smri_thick_cdk_rrmdfrlh | -0.030 | -0.048 | -0.012 | -3.324 | 6240.011 | 0.001 | 0.021 | 0.549 | 0.046 |
| smri_thick_cdk_sufrlh | -0.028 | -0.044 | -0.011 | -3.340 | 6259.082 | 0.001 | 0.021 | 0.642 | 0.047 |
| smri_thick_cdk_supllh | -0.015 | -0.033 | 0.003 | -1.638 | 6226.643 | 0.102 | 0.411 | 0.574 | 0.023 |
| smri_thick_cdk_sutmlh | -0.010 | -0.024 | 0.004 | -1.365 | 6240.346 | 0.172 | 0.469 | 0.717 | 0.019 |
| smri_thick_cdk_smlh | -0.008 | -0.025 | 0.010 | -0.870 | 6216.765 | 0.385 | 0.623 | 0.585 | 0.012 |
| smri_thick_cdk_frpolelh | -0.018 | -0.035 | 0.000 | -2.000 | 5999.385 | 0.046 | 0.258 | 0.594 | 0.028 |
| smri_thick_cdk_tmpolelh | 0.000 | -0.023 | 0.022 | -0.032 | 6160.936 | 0.974 | 0.974 | 0.339 | 0.000 |
| smri_thick_cdk_trvtmlh | 0.005 | -0.009 | 0.020 | 0.702 | 6156.239 | 0.483 | 0.684 | 0.711 | 0.010 |
| smri_thick_cdk_insulalh | -0.013 | -0.035 | 0.008 | -1.211 | 6292.177 | 0.226 | 0.498 | 0.367 | 0.017 |
| smri_thick_cdk_banksstsrh | -0.009 | -0.021 | 0.003 | -1.467 | 5718.859 | 0.142 | 0.421 | 0.805 | 0.021 |
| smri_thick_cdk_cdacaterh | 0.005 | -0.008 | 0.018 | 0.755 | 5562.030 | 0.451 | 0.678 | 0.780 | 0.011 |
| smri_thick_cdk_cdmdfrrh | -0.013 | -0.031 | 0.006 | -1.330 | 6292.698 | 0.184 | 0.480 | 0.538 | 0.019 |
| smri_thick_cdk_cuneusrh | 0.003 | -0.010 | 0.017 | 0.473 | 6151.531 | 0.636 | 0.801 | 0.757 | 0.007 |
| smri_thick_cdk_ehinalrh | -0.024 | -0.044 | -0.004 | -2.305 | 6049.115 | 0.021 | 0.192 | 0.451 | 0.032 |
| smri_thick_cdk_fusiformrh | -0.003 | -0.019 | 0.013 | -0.383 | 6150.110 | 0.702 | 0.838 | 0.669 | 0.005 |
| smri_thick_cdk_ifplrh | -0.008 | -0.025 | 0.008 | -0.988 | 6188.831 | 0.323 | 0.575 | 0.621 | 0.014 |
| smri_thick_cdk_iftmrh | -0.015 | -0.031 | 0.001 | -1.864 | 6171.690 | 0.062 | 0.303 | 0.671 | 0.026 |
| smri_thick_cdk_ihcaterh | -0.002 | -0.014 | 0.011 | -0.287 | 5961.867 | 0.774 | 0.877 | 0.802 | 0.004 |
| smri_thick_cdk_loccrh | -0.002 | -0.016 | 0.011 | -0.329 | 6276.778 | 0.742 | 0.870 | 0.745 | 0.005 |
| smri_thick_cdk_lobfrrh | -0.016 | -0.036 | 0.004 | -1.579 | 6266.879 | 0.114 | 0.411 | 0.446 | 0.022 |
| smri_thick_cdk_lingualrh | 0.005 | -0.008 | 0.019 | 0.741 | 6217.215 | 0.459 | 0.678 | 0.748 | 0.010 |
| smri_thick_cdk_mobfrrh | -0.002 | -0.022 | 0.017 | -0.248 | 6281.171 | 0.804 | 0.897 | 0.456 | 0.003 |
| smri_thick_cdk_mdtmrh | -0.018 | -0.034 | -0.003 | -2.280 | 6279.257 | 0.023 | 0.192 | 0.670 | 0.032 |
| smri_thick_cdk_parahpalrh | -0.001 | -0.014 | 0.012 | -0.151 | 6013.160 | 0.880 | 0.947 | 0.781 | 0.002 |
| smri_thick_cdk_paracnrh | -0.010 | -0.027 | 0.006 | -1.274 | 6296.825 | 0.203 | 0.498 | 0.656 | 0.018 |
| smri_thick_cdk_parsopcrh | -0.001 | -0.016 | 0.014 | -0.137 | 6217.719 | 0.891 | 0.947 | 0.706 | 0.002 |
| smri_thick_cdk_parsobisrh | -0.009 | -0.025 | 0.006 | -1.213 | 6218.757 | 0.225 | 0.498 | 0.687 | 0.017 |
| smri_thick_cdk_parstgrisrh | -0.017 | -0.035 | 0.001 | -1.876 | 6234.332 | 0.061 | 0.303 | 0.585 | 0.026 |
| smri_thick_cdk_periccrh | -0.005 | -0.020 | 0.010 | -0.650 | 6284.263 | 0.516 | 0.702 | 0.693 | 0.009 |
| smri_thick_cdk_postcnrh | -0.007 | -0.022 | 0.008 | -0.903 | 6402.712 | 0.366 | 0.608 | 0.675 | 0.013 |
| smri_thick_cdk_ptcaterh | 0.009 | -0.005 | 0.023 | 1.261 | 6216.181 | 0.207 | 0.498 | 0.744 | 0.018 |
| smri_thick_cdk_precnrh | -0.015 | -0.033 | 0.004 | -1.552 | 6250.778 | 0.121 | 0.411 | 0.534 | 0.022 |
| smri_thick_cdk_pcrh | 0.001 | -0.014 | 0.015 | 0.066 | 6257.679 | 0.947 | 0.972 | 0.705 | 0.001 |
| smri_thick_cdk_rracaterh | -0.003 | -0.021 | 0.016 | -0.289 | 6271.873 | 0.772 | 0.877 | 0.557 | 0.004 |
| smri_thick_cdk_rrmdfrrh | -0.026 | -0.045 | -0.008 | -2.807 | 6260.673 | 0.005 | 0.085 | 0.530 | 0.039 |
| smri_thick_cdk_sufrrh | -0.019 | -0.036 | -0.002 | -2.146 | 6300.165 | 0.032 | 0.227 | 0.586 | 0.030 |
| smri_thick_cdk_suplrh | -0.007 | -0.025 | 0.012 | -0.706 | 6247.827 | 0.480 | 0.684 | 0.560 | 0.010 |
| smri_thick_cdk_sutmrh | -0.006 | -0.021 | 0.009 | -0.809 | 6214.611 | 0.418 | 0.662 | 0.715 | 0.011 |
| smri_thick_cdk_smrh | -0.021 | -0.038 | -0.004 | -2.432 | 6128.249 | 0.015 | 0.171 | 0.596 | 0.034 |
| smri_thick_cdk_frpolerh | -0.015 | -0.033 | 0.003 | -1.654 | 5964.990 | 0.098 | 0.411 | 0.582 | 0.023 |
| smri_thick_cdk_tmpolerh | -0.038 | -0.060 | -0.015 | -3.308 | 6223.136 | 0.001 | 0.021 | 0.333 | 0.046 |
| smri_thick_cdk_trvtmrh | 0.008 | -0.007 | 0.023 | 1.008 | 5860.208 | 0.313 | 0.575 | 0.691 | 0.014 |
| smri_thick_cdk_insularh | 0.011 | -0.011 | 0.032 | 0.995 | 6284.906 | 0.320 | 0.575 | 0.379 | 0.014 |

CI, confidence interval

The codes of the brain structures can be checked at the ABCD study website: https://nda.nih.gov/data_structure.html?short_name=abcd_smrip101
